# Multimorbidity, health care utilization and costs in an elderly community-dwelling population: a claims data based observational study

**DOI:** 10.1186/s12913-015-0698-2

**Published:** 2015-01-22

**Authors:** Caroline Bähler, Carola A Huber, Beat Brüngger, Oliver Reich

**Affiliations:** Department of Health Sciences, Helsana Insurance Group, P.O. Box, 8081, Zürich, Switzerland

**Keywords:** Health care utilization, Health care costs, Multimorbidity, Claims data

## Abstract

**Background:**

Chronic conditions and multimorbidity have become one of the main challenges in health care worldwide. However, data on the burden of multimorbidity are still scarce. The purpose of this study is to examine the association between multimorbidity and the health care utilization and costs in the Swiss community-dwelling population, taking into account several sociodemographic factors.

**Methods:**

The study population consists of 229'493 individuals aged 65 or older who were insured in 2013 by the Helsana Group, the leading health insurer in Switzerland, covering all 26 Swiss cantons. Multimorbidity was defined as the presence of two or more chronic conditions of a list of 22 conditions that were identified using an updated measure of the Pharmacy-based Cost Group model. The number of consultations (total and divided by primary care physicians and specialists), the number of different physicians contacted, the type of physician contact (face-to-face, phone, and home visits), the number of hospitalisations and the length of stay were assessed separately for the multimorbid and non-multimorbid sample. The costs (total and divided by inpatient and outpatient costs) covered by the compulsory health insurance were calculated for both samples. Multiple linear regression modelling was conducted to adjust for influencing factors: age, sex, linguistic region, purchasing power, insurance plan, and nursing dependency.

**Results:**

Prevalence of multimorbidity was 76.6%. The mean number of consultations per year was 15.7 in the multimorbid compared to 4.4 in the non-multimorbid sample. Total costs were 5.5 times higher in multimorbid patients. Each additional chronic condition was associated with an increase of 3.2 consultations and increased costs of 33%. Strong positive associations with utilization and costs were also found for nursing dependency. Multimorbid patients were 5.6 times more likely to be hospitalised. Furthermore, results revealed a significant age-gender interaction and a socioeconomic gradient.

**Conclusions:**

Multimorbidity is associated with substantial higher health care utilization and costs in Switzerland. Quantified data on the current burden of multimorbidity are fundamental for the management of patients in health service delivery systems and for health care policy debates about resource allocation. Strategies for a better coordination of multimorbid patients are urgently needed.

**Electronic supplementary material:**

The online version of this article (doi:10.1186/s12913-015-0698-2) contains supplementary material, which is available to authorized users.

## Background

In view of the growing number of elderly individuals and the expected increase of patients with two or more chronic diseases, and also due to the increased availability of new therapies and advanced medical technologies, the need of health care especially in the elderly general population is becoming a major public health concern. The high prevalence of multiple chronic conditions in elderly (usually exceeding 60%) and the associated disproportional direct health care costs have been exhibited in previous studies [1-5]. In addition, multimorbidity – usually defined as the presence of two or more chronic conditions – is related to a higher risk of care dependency [6], which in turn causes further expenditures.

More particularly, multimorbidity was associated with more than twice as many contacts per year with physicians, whereby the number of contacts as well as the number of different physicians contacted increased steadily with each additional chronic condition [7]. Beyond that, the number of diagnosis groups or comorbidities was a main predictor of the number of hospital admissions in elderly people [8,9]. Multimorbidity was further associated with higher need for specialised care and higher referrals to specialised care, respectively [10-12]. However, differences exist depending on the underlying health care system and the function of primary care physicians (e.g. gatekeeper). In contrast, while a higher use of medications was most strongly predicted by more medical diagnoses, physician contact was only weakly associated with medical diagnosis in a previous German study [13].

Findings from the U.S. have shown that as much as three fourth of the total health care costs are related to the treatment of chronic conditions [14]. In Switzerland, data concerning the total costs for the treatment of chronic conditions are lacking. According to recent estimates the direct health care costs for seven main chronic diseases amounted to CHF 33.1 billion CHF (US$ 35.5 billion, exchange rate: September, 10^th^, 2014) in 2011, corresponding to 51.1% of the total health care costs [15]. The Swiss Federal Council has listed the increase in chronic diseases as the major challenge for the upcoming eight years in his report on Health 2020 [16].

To date, little is also known about the concept of multimorbidity. Apart from age and gender, socioeconomic status has been shown to be an important influencing factor with regard to prevalence rates as well as health care utilization rates [3,17-19]. Even though in the majority of studies multimorbidity was shown to be a strong predictor for higher health care utilization rates and health care costs, especially in the elderly population, data are still scarce and inconsistent in terms of the impact of predisposing and influencing factors [10]. Notably, there is only little data in Europe and hardly any in Switzerland on the association between multimorbidity and health care utilization as well as health care costs in the elderly population [10]. Furthermore, in Switzerland, no data on the aggregated costs of multiple chronic conditions exist.

### Aim of the study

The purpose of this study is to examine the relationship between multimorbidity and the number of consultations by physicians, the number of hospitalisations, as well as the health care costs in patients with multimorbidity in the Swiss community-dwelling population, accounting for a variety of influencing, sociodemographic factors. Findings should provide further information on the burden of multimorbidity and on the health care utilization patterns in patients with multiple chronic conditions in Switzerland. Results may bring about important implications for research and policy concerning future demands for health services in elderly patients.

## Methods

### Study design

All analyses conducted for the purpose of this study were based on claims data of the Helsana Group, the leading health insurer in Switzerland, covering all 26 Swiss cantons. Data of the year 2013 and, for comparison reasons, of the year 2012 were collected. An estimated percentage of 3% of the invoices are not sent to the health insurance, mainly because they are paid directly by the patients. These data could not be included in the analyses.

In compliance with the Swiss Federal Law on data protection, all data were anonymized and de-identified to protect the privacy of patients, physicians, and hospitals. Because the data were retrospective, pre-existing, and de-identified, this study was exempted from ethics committee approval. The study protocol was approved by the Helsana Group.

### Study population

The study population was extracted from a dataset of 266'925 individuals aged 65 or older who were insured by the Helsana Group in the year 2013. During the study year period from January 1, 2013 to December 31, 17'151 (6.4%) dropped out of the Helsana Group and were therefore excluded. Additionally, 899 (0.3%) individuals who died in 2013, 18'547 (6.9%) nursing home residents and 835 (0.3%) individuals with missing data (e.g. individuals living abroad) were excluded from the study. Home residents had to be excluded because detailed and complete information on medications are lacking in this group of insurants.

### Measures

#### Chronic conditions and multimorbidity

Chronic conditions were identified using an updated measure of the Pharmacy-based Cost Group (PCG) model by Huber et al. [20], which is based on the Anatomical Therapeutic Chemical (ATC) classification system. Twenty-two different chronic conditions were distinguished (Acid related disorders, Bone diseases (osteoporosis), Cancer, Cardiovascular diseases (incl. hypertension), Dementia, Diabetes mellitus, Epilepsy, Glaucoma, Gout/Hyperuricemia, HIV, Hyperlipidemia, Intestinal inflammatory diseases, Iron deficiency anemia, Migraines, Pain, Parkinson’s disease, Psychological disorders (sleep disorder, depression), Psychoses, Respiratory illness (asthma, COPD), Rheumatologic conditions, Thyroid disorders, and Tuberculosis). The use of administrative data of treated conditions was taken because clinical diagnoses are not available from administrative data in the outpatient setting in Switzerland. Multimorbidity was defined as the presence of two or more chronic conditions in one person [3-5,10,11].

### Health care utilization

The number of consultations in 2013 (divided by primary care physicians and specialists), the type of physician contact (including face-to-face consultations, phone consultations, and home visits), as well as the number of different physicians contacted were derived from the dataset. Face-to-face-consultations were defined as a visit with direct physician contact. Besides, the number of hospitalisations, if any, and the mean length of hospital stay (divided by acute hospitals, psychiatric hospitals, and others, e.g. rehabilitations) were calculated. Furthermore, the number of different ATCs was assessed. Because the use of health care resources in the preceding year has been shown to be highly predictive, the number of consultations in the year 2012 was also taken into account in the analyses.

### Health care costs

The total costs covered by the compulsory health insurance were calculated for each patient. Accident-related costs are defined as costs that are related to a sudden, unintended and harmful impact of a unusual external factor on the human body. As these accident-related costs are financed separately from disease-related costs in the Swiss health care system, these costs were excluded. Disease-related costs (arising from impairment to physical or mental health, but not as a result of an accident) were further divided into inpatient (acute hospitals, psychiatric hospitals, and others) and outpatient (primary care physicians, specialists, home care nursing services, hospital outpatient visits, and others) costs. The category ‘other inpatient costs’ encompassed costs of inpatient rehabilitation centres, acute and transitional care services and of emergency transport services. Hospital outpatient visit costs comprised costs of physicians from ambulatories, outpatient clinics and walk-in-clinics. Costs of paramedical practitioners (e.g. chiropractors or physiotherapists), laboratory tests and medical devices were assigned to other outpatient costs. Medication costs as part of the total costs included those of medications which were disposed directly at the pharmacy as well, unless they were paid directly by the patients. Finally, data on the total health care costs of the preceding year were included in the analyses.

### Sociodemographic factors

The following sociodemographic factors were assessed: age, gender, linguistic region (German, French, Italian or Rhaeto-Romanic), as well as specific characteristics of the individuals’ health insurance plan. Health insurance is mandatory in Switzerland and mainly financed by a premium each resident has to pay. The height of the premium can be decreased by the insurant by choosing a higher deductible. Deductible classes vary between Swiss francs (CHF) 300 and CHF 2500 per year, which equals approximately US$ 322 and US$ 2681, respectively. The variable concerning higher deductible classes was dichotomised into ‘no’ if the deductible was chosen to be CHF 300 or CHF 500 or ‘yes’ if the deductible was CHF 1000, CHF 1500, CHF 2000 or CHF 2500. Furthermore, insurants can choose to pursue a managed care model (including family doctor models or telemedicine models) in order to further reduce the height of the premium, and to take out an additional coverage for accidents. Socioeconomic situation was defined by using a proxy of purchasing power of the corresponding zip code [17]. The standardised data in the present study are based on the statistical report of the international polling institute GFK and range from −1.8 (zip codes with lowest purchasing power) to 6.6 (zip codes with highest purchasing power). For comparison reasons, the data on purchasing power were divided into quintiles. Finally, a binary variable on whether or not individuals required home care nursing services was also included in the analyses.

### Statistical analysis

Based on claims data, descriptive statistics were used to show differences of the characteristics between individuals with and without multiple chronic conditions using fisher’s exact test, chi-squared test and Wilcoxon rank sum test. The number of consultations, the type of contacts, and the number of different physicians contacted as well as the health care costs are presented for both samples. Figures are provided of the number of consultations (total and divided by primary care physician and specialist) per additional chronic condition using boxplots, and on the health care costs (total and divided by inpatient and outpatient costs) per additional chronic condition using a strip chart. Linear regression lines and LOESS (Local Polynomial Regression Fitting) smoother were added to the strip chart.

Linear regression analyses were performed to evaluate the association between the number of consultations or health care costs and the number of chronic conditions. The number of consultations and the health care costs served as dependent variables and the number of chronic conditions as independent variables. Moreover, multiple linear regression modelling was conducted to adjust for influencing factors: age, gender, linguistic region, purchasing power, insurance plan, and nursing dependency. Separate models for consultations by primary care physicians and specialists as well as for inpatient and outpatient costs have been built. As health care utilization and costs are strongly age-dependent, age groups were built. The gender related difference in the age-dependency of the utilization and costs has been modelled with interaction terms. The influence of each age group is shown separately for men and women. To account for the non-linear relationship between predictors and response variable, the log-transformation was applied. Model appropriateness was assessed using Tukey-Anscombe-Plots and normal Q-Q plots. The quality of the model was quantified by R2. All analyses were conducted using R statistics, version 3.1.0.

## Results

Of the 229'493 individuals, 175'752 (76.6%) had two or more chronic conditions. The mean (± SD) age of the total sample was 74.9 (±7.2; 75.6 ± 7.2 in the multimorbid compared to 72.7 ± 6.7 in the non-multimorbid sample). The proportion of individuals with multimorbidity increased from 74.8% in communities with the highest purchasing power to 77.4% in communities with the lowest purchasing power. Descriptive statistics of the characteristics of multimorbid versus non-multimorbid individuals are shown in Table 1.

**Table 1:** **Baseline characteristics of an elderly population (≥65 years of age) with and without multimorbidity**

**Baseline characteristics n (%)**	**Total study sample (n = 229493)**	**Non-multi-morbid (n = 53741)**	**Multi-morbid (n = 175752)**	***Sign.***
Gender				***
Male	98'124 (42.8)	24'936 (46.4)	73'188 (41.6)	
Female	131'369 (57.2)	28'805 (53.6)	102'564 (58.4)	
Age group				***
65-69	66'190 (28.9)	22'156 (41.2)	44'034 (25.1)	
70-74	56'073 (24.4)	14'157 (26.3)	41'916 (23.8)	
75-79	45'720 (19.9)	8'553 (15.9)	37'167 (21.1)	
80-84	34'803 (15.2)	5'137 (9.6)	29'666 (16.9)	
85+	26'707 (11.6)	3'738 (7.0)	22'969 (13.1)	
Linguistic region				***
German	174'171 (75.9)	43'624 (81.2)	130'547 (74.3)	
French	34'864 (15.2)	6'413 (11.9)	28'451 (16.2)	
Italian	20'024 (8.7)	3'592 (6.7)	16'432 (9.3)	
Rhaeto-Romanic	434 (0.2)	112 (0.2)	322 (0.2)	
Insurance plan				
Managed care	94'421 (41.1)	24'316 (45.2)	70'105 (39.9)	***
Higher deductible	30'419 (13.3)	17'212 (32.0)	13'207 (7.5)	***
Accident coverage	224'891 (98.0)	51'900 (96.6)	172'991 (98.4)	***
Home care nursing dependency	19'549 (8.5)	867 (1.6)	18'682 (10.6)	***
Chronic conditions [mean, (SD)]	3.29 (2.19)	0.47 (0.50)	4.15 (1.73)	***
Purchasing power				***
1 (high)	45'653 (19.9)	11'517 (21.4)	34'136 (19.4)	
2	45'709 (19.9)	11'175 (20.8)	34'727 (19.8)	
3	45'902 (20.0)	10'370 (19.3)	35'339 (20.1)	
4	45'924 (20.0)	10'285 (19.1)	36'020 (20.5)	
5 (low)	46'305 (20.2)	10'394 (19.3)	35'530 (20.2)	

***p-value <0.001 **p-value <0.01 *p-value <0.05.

The mean (± SD) number of chronic conditions in the study population was slightly higher in women than men (3.4 (±2.2) vs 3.1 (±2.1), p < 0.001), and increased with age (from 2.7 (±2.1) in the youngest to 3.8 (±2.0) in the oldest age group). The median number amounted to 3 chronic conditions (range: 0–13, interquartile range: 3) in the total study population.

### Health care utilization

Overall, the number of individuals demanding health care was significantly higher in the multimorbid compared to the non-multimorbid-sample (Table 2). Almost all (98.5%) patients with multimorbidity had at least one consultation in the year 2013 compared with 68.7% in the non-multimorbid-sample. The multimorbid-sample was almost twice as likely to have had a consultation by a primary care physician and by a specialist, respectively. Furthermore, approximately 22% of the multimorbid patients were seen by more than one primary care physician in 2013 and over 54% consulted more than one specialist over the same time period. Moreover, multimorbid patients were 5.6 times more likely to be hospitalised compared to the non-multimorbid sample.

**Table 2:** **Number of elderly individuals (≥65 years of age) with and without multimorbidity demanding health care in 2013**

**Health care utilization n (%)**	**Total study sample (n = 229493)**	**Non-multi-morbid (n = 53741)**	**Multi-morbid (n = 175752)**	***Sign.***
Individuals with consultation	209'962 (91.5)	36'922 (68.7)	173'040 (98.5)	***
By primary care physicians	185'035 (80.6)	27'397 (51.0)	157'638 (89.7)	***
By specialists	165'178 (72.0)	25'834 (48.1)	139'344 (79.3)	***
Hospital outpatient visits	108'502 (47.3)	12'168 (22.6)	96'334 (54.8)	***
By different primary care physicians	41'601 (18.1)	3'494 (6.5)	38'107 (21.7)	***
By different specialists	108'187 (47.1)	13'029 (24.2)	95'158 (54.1)	***
Individuals with physician contact				
Face-to-face consultations	203'328 (88.6)	34'129 (63.5)	169'199 (96.3)	***
Phone consultations	55'174 (24.0)	4'748 (8.8)	50'426 (28.7)	***
Home visits	14'059 (6.1)	654 (1.2)	13'405 (7.6)	***
Individuals with hospitalisation	40'556 (17.7)	2'091 (3.9)	38'465 (21.9)	***

***p-value <0.001 **p-value <0.01 *p-value <0.05.

**Figure 1:**
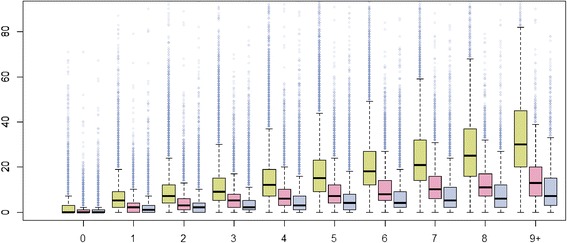
**The number of consultations per year according to the number of chronic conditions in an elderly population (≥65 years of age) in Switzerland, 2013.** Green boxplots: Total number of consultations; red boxplots: Number of consultations by primary care physicians; blue boxplots: Number of consultations by specialists.

The mean number of consultations per year amounted to 15.7 (median: 10) in the multimorbid-sample compared to 4.4 (median: 2) in the non-multimorbid-sample (Table 3). In women, the corresponding figures were slightly higher than in men (16.0 and 4.6 versus 15.3 and 4.1; results not shown). Moreover, we found an increase of 3.2 consultations per each additional chronic condition. The increase in the number (and distribution) of consultations per each additional chronic condition is illustrated in Figure 1. An increase can be seen in primary care physicians as well as in specialists, even though to a lesser extent in the latter (Figure 1).

**Table 3:** **Health care utilization in an elderly population (≥65 years of age) with and without multimorbidity**

**Health care utilization mean number (SD) per year**	**Total study sample (n = 229493)**	**Non-multi-morbid (n = 53741)**	**Multi-morbid (n = 175752)**	***Sign.***
Consultations (total)	13.1 (13.7)	4.4 (6.3)	15.7 (14.2)	***
By primary care physicians	6.1 (6.7)	1.9 (3.1)	7.4 (7.0)	***
By specialists	4.3 (6.4)	1.8 (3.4)	5.1 (6.8)	***
Hospital outpatient visits	2.7 (7.9)	0.7 (3.0)	3.2 (8.8)	***
Physician contacts				
Face-to-face consultations	8.1 (7.6)	2.9 (4.0)	9.7 (7.8)	***
Phone consultations	0.5 (1.6)	0.1 (0.6)	0.7 (1.8)	***
home visits	0.2 (1.6)	0.0 (0.6)	0.3 (1.8)	***
Different ATCs^1^	10.7 (8.3)	2.4 (2.9)	13.3 (7.8)	***
Different physicians contacted				
Total	2.9 (2.1)	1.5 (1.6)	3.3 (2.1)	***
Different primary care physicians	1.1 (0.7)	0.6 (0.6)	1.2 (0.7)	***
Different specialists	1.8 (1.9)	0.9 (1.3)	2.1 (1.9)	***
Hospitalisations^2^	1.5 (0.9)	1.2 (0.5)	1.5 (0.9)	***
Length of hospital stay^2^	13.9 (20.9)	7.9 (18.3)	14.3 (21.0)	***
Acute hospitals	11.0 (16.0)	6.0 (9.1)	11.3 (16.2)	***
Psychiatric hospitals	0.9 (9.6)	0.7 (9.6)	1.0 (9.7)	
Others (e.g. rehabilitations)	2.0 (8.6)	1.2 (12.6)	2.0 (8.3)	***

***p-value <0.001 **p-value <0.01 *p-value <0.05.

^1^ATC = Anatomical Therapeutic Chemical.

^2^only in patients with at least one hospitalisation.

Likewise, 3.3 different physicians were contacted in the multimorbid-sample compared to 1.5 in the non-multimorbid-sample (Table 3) in the year 2013. Although the number of hospitalisations was not different in both samples (1.5 versus 1.2), the number of days in the hospital was twice as high in multimorbid compared to non-multimorbid individuals (when looking at patients with at least one hospitalisation). The most frequent type of physician contacts was face-to-face consultations with a statistically significant difference between the two samples.

In the multiple linear regression model on the total number of consultations per year, the two main predictors were the number of consultations in the preceding year and the number of chronic conditions (Table 4). Each increase of a chronic condition resulted in an increase of consultations of almost 20%. Age was significantly associated with a higher number of consultations per year. This held true for the age groups of 70–74 years, 75–79 years and of 80–84 years, compared with individuals aged 65 to 69 years. However, significant interactions were found between age group and gender. While the number of consultations increased by 6.7%, 8.4% and 9.1% with increasing age in men, the corresponding figures revealed a declining increase in women of 7.4%, 4.4% and 0.4%, although results were not significantly different for this last age group (of women aged 80 to 84 years compared to 65 to 69 years). In individuals aged 85 or older, results exhibited a significant decrease of consultations of 5.1% in women, whereas an increase of 7.6% was found in men of the same age. Demanding home care nursing services and being in a managed care model were significantly associated with higher number of consultations as well: the increase amounted to 11.5% and 2.9%, respectively. In contrast, choosing a higher deductible and living in the French- or Italian-speaking part of Switzerland (as compared with the German-speaking part) were associated with lower numbers of consultations. There was a small but significant socioeconomic gradient: the lower the purchasing power of a community, the lower the number of consultations. The decrease was 7.8% in patients living in a community with the lowest compared to the highest purchasing power. Accident coverage did not significantly influence the outcome.

**Table 4:** **Multiple linear regression model on the total number of consultations per year in an elderly population (≥65 years of age) (n = 229493)**

**Total number of consultations**		
	**B (95% CI)**	***Sign.***
Age group by male gender		
65-69 (male)	1.000	
70-74 (male)	1.067 (1.055 - 1.079)	***
75-79 (male)	1.084 (1.071 - 1.097)	***
80-84 (male)	1.091 (1.076 - 1.106)	*
85+ (male)	1.076 (1.059 - 1.093)	***
Age group by female gender		
65-69 (female)	1.000	
70-74 (female)	1.074 (1.051 - 1.097)	***
75-79 (female)	1.044 (1.021 - 1.069)	***
80-84 (female)	1.004 (0.979 - 1.029)	
85+ (female)	0.949 (0.923 - 0.975)	***
Number of chronic conditions	1.191 (1.189 - 1.193)	***
Linguistic region		
German	1.000	
French	0.940 (0.933 - 0.948)	***
Italian	0.979 (0.969 - 0.989)	***
Rhaeto-Romanic	0.988 (0.927 - 1.052)	
Purchasing power		
1 (high)	1.000	
2	0.975 (0.967 - 0.984)	***
3	0.958 (0.949 - 0.966)	***
4	0.947 (0.939 - 0.955)	***
5 (low)	0.922 (0.914 - 0.931)	***
Deductible class	0.895 (0.887 - 0.903)	***
Managed care	1.029 (1.023 - 1.035)	***
Accident coverage	1.001 (0.981 - 1.021)	
Nursing dependency	1.115 (1.103 - 1.126)	***
Number of consultations in 2012	1.588 (1.583 - 1.593)	***
R^2^	.565	

***p-value <0.001 **p-value <0.01 *p-value <0.05.

When looking at the number of consultations by primary care physicians and by specialists separately, the influence of the main predictors remained almost the same (see Additional files 1 and 2 for detailed information).

### Health care costs

Overall, 50.8% of the total costs accounted for outpatient services, 24.1% for inpatient services, and 25.1% accounted for medication costs.

**Figure 2:**
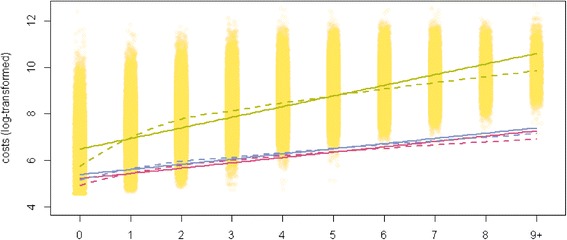
**Health care costs per year according to the number of chronic conditions in an elderly population (≥65 years of age) in Switzerland, 2013.** Green lines: Total health care costs; red lines: Health care costs by primary care physicians; blue lines: Health care costs by specialists. Straight lines = linear regression lines; Dashed lines = LOESS smoother.

Mean total health care costs were 5.5 times higher in the multimorbid patients compared to those with none or only one chronic condition (Table 5). Considerable mean higher costs in multimorbid patients were found for both subgroups: for inpatient and outpatient costs. As far as inpatient costs are concerned, the differences in costs were highest in acute hospitals compared to psychiatric or other hospital settings (e.g. rehabilitations). As for the outpatient costs, relative differences were notably found for home care nursing services and hospital outpatient visits. Relative differences in mean costs between multimorbid versus non-multimorbid individuals were higher for primary care physicians compared to specialists, even though the mean costs for specialists were almost twice as high in both samples. Each additional chronic condition was associated with increased costs of CHF 2222 (US$ 2383) per year. Figure 2 illustrates the log-transformed total health care costs, the costs by primary care physicians and the costs by specialists per year according to the number of chronic conditions. While the costs seem to increase almost linearly in individuals suffering from four to six chronic conditions, the LOESS smoother show lower than expected costs in individuals with no chronic condition and in those with seven or more chronic conditions.

**Table 5:** **Health care costs (in Swiss francs) in an elderly population (≥65 years of age) with and without multimorbidity**

**Health care costs mean (SD) per year**	**Total study sample (n = 229493)**	**Non-multi-morbid (n = 53741)**	**Multi-morbid (n = 175752)**	***Sign.***
Health care costs				
Total	6711 (11325)	1515 (3774)	8301 (12342)	***
Inpatient	1616 (5847)	238 (2264)	2038 (6505)	***
Outpatient	3410 (5678)	983 (2185)	4152 (6187)	***
Inpatient costs				
Acute hospitals	1468 (5345)	213 (1886)	1852 (5968)	***
Psychiatric hospitals	48 (1142)	10 (597)	60 (1262)	***
Others (e.g. rehabilitations)	100 (1052)	15 (623)	126 (1150)	***
Outpatient costs				
Primary care physicians	479 (598)	146 (280)	581 (632)	***
Specialists	849 (1397)	322 (735)	1010 (1508)	***
Home care nursing services	337 (2202)	60 (910)	421 (2459)	***
Hospital outpatient visits	905 (3592)	228 (1333)	1112 (4015)	***
Others (e.g. paramedical visits)	840 (1704)	227 (620)	1028 (1877)	***

***p-value <0.001 **p-value <0.01 *p-value <0.05.

In the multiple linear regression model on the total health care costs per year, the main predictors were nursing dependency and total health care costs in the preceding year, followed by the number of chronic conditions (Table 6). Costs increased by 32.6% for each additional chronic condition and almost doubled in patients demanding home care nursing services. Furthermore, results revealed significant interactions between age and gender: total costs increased by 9-10% in men aged 70 to 84 years, respectively, when compared to men aged 65 to 69 years. A slighter increase of 5.7% was found for the oldest age group. Overall, female gender was associated with lower costs, whereby costs decreased with increasing age. This held true for women aged 75 and older. The lowest total health care costs were therefore found in women aged 85 and older, showing a decrease of 12.1% compared to women of the youngest age group. Again, there was a small but significant socioeconomic gradient. The lower the purchasing power of a community, the lower turned out the costs of the patient: the decrease made up 3.4%, 6.6%, 9.5% and 13.4% with each decrease in the purchasing power quintile, compared to patients living in a community with the highest purchasing power. Compared with the German-speaking part, the Italian-speaking part of Switzerland was associated with slightly lower costs of 2.3%. No significant differences were found for the French- and the Rhaeto-Romanic-speaking part. Choosing a higher deductible was significantly and negatively associated with total costs, amounting to a decrease of almost one fifth, whereas no association was found between the total costs and being in a managed care model. Accident coverage did not significantly influence the results either.

**Table 6:** **Multiple linear regression model on the total health care costs per year in an elderly population (≥65 years of age) (n = 229493)**

**Total health care costs**		
	**B (95% CI)**	***Sign.***
Age group by male gender		
65-69 (male)	1.000	
70-74 (male)	1.086 (1.070 - 1.102)	***
75-79 (male)	1.103 (1.085 - 1.120)	***
80-84 (male)	1.090 (1.071 - 1.110)	***
85+ (male)	1.057 (1.036 - 1.080)	***
Age group by female gender		
65-69 (female)	1.000	
70-74 (female)	1.011 (0.983 - 1.040)	
75-79 (female)	0.967 (0.939 - 0.996)	*
80-84 (female)	0.920 (0.891 - 0.951)	***
85+ (female)	0.879 (0.848 - 0.912)	***
Number of chronic conditions	1.326 (1.324 - 1.329)	***
Linguistic region		
German	1.000	
French	0.991 (0.981 - 1.001)	
Italian	0.977 (0.964 - 0.990)	***
Rhaeto-Romanic	0.994 (0.915 - 1.080)	
Purchasing power		
1 (high)	1.000	
2	0.966 (0.955 - 0.977)	***
3	0.934 (0.923 - 0.945)	***
4	0.905 (0.895 - 0.916)	***
5 (low)	0.866 (0.856 - 0.876)	***
Deductible class	0.817 (0.808 - 0.826)	***
Managed care	0.998 (0.991 - 1.006)	
Accident coverage	1.000 (0.974 - 1.026)	
Nursing dependency	1.980 (1.952 - 2.007)	***
Total health care costs in 2012	1.464 (1.459 - 1.468)	***
R^2^	.617	

***p-value <0.001 **p-value <0.01 *p-value <0.05.

When looking at inpatient and outpatient costs separately, the extent and direction of the influencing factors remained similar. Further information is shown in the Additional files 3 and 4.

## Discussion

Overall, we found a high prevalence of multimorbidity in this sample of a Swiss community-dwelling elderly population, and considerable variations in health care utilization and health care costs in individuals with and without multiple chronic conditions.

The prevalence of multimorbidity amounted to 76.6%. Similar prevalence rates in the elderly (exceeding 60%) have been shown in varies studies [2,4]. In the U.S. study, 64.7% of Medicare fee-for-service beneficiaries aged 65 or older had multiple chronic conditions [2], accounting for 95% of the total health care expenditures. The result is also comparable to the result of a previous study conducted in a Swiss medical division of a tertiary care teaching hospital, demonstrating a prevalence rate of 61.0% in patients 65 or older [21]. In the German study by van den Bussche et al. [7], 58.9% of the elderly population were considered multimorbid according to their definition; multimorbidity was defined as the presence of three or more out of a list of 46 different chronic conditions. Our estimate of the prevalence of multimorbidity is much higher than that of a Swiss study conducted in 2010/2011 including 3800 persons revealing a prevalence rate of 25.8% in persons aged 65 or older [22]. The great difference in the total proportion of multimorbid patients might be due to the fact, that their results were based on self-report and the number of the listed chronic conditions was 12. In line with our results, higher prevalence rates were found in women in their study. Consistent with these results, a higher prevalence rate of multimorbidity in women compared to men has been reported in international studies [3,23,24].

### Health care utilization

The mean number of consultations per year amounted to 15.7 in the multimorbid compared to 4.4 in the non-multimorbid sample, with an increase of 3.2 consultations per each additional chronic condition. Of the patients with multiple chronic conditions, every fifth consulted more than one primary care physician, and more than half consulted more than one specialist in the year 2013. The proportion of patients who had at least one hospitalisation in this same year was 21.9% in the multimorbid versus 3.9% in the non-multimorbid sample. The length of stay doubled in the presence of multimorbidity.

Our results of increased numbers of consultations with increasing number of chronic conditions are in line with previous analyses [4,7,10,11]. In a Spanish study [17], the median (interquartile range) number of primary care visits in multimorbid versus non-multimorbid individuals was 8 (4–14) and 1 (0–4), respectively, which resembles our findings with median numbers of consultations of 10 and 2, respectively. In the Dutch survey by van Oostrom et al. [11], the mean number of contacts per year was significantly higher in patients with multimorbidity compared to patients with only one or none chronic disease (18.3 vs. 11.7 and 6.1 contacts, respectively). These numbers are higher than those found in our study, even though they only looked at contacts in the general practice. In a German study [7], corresponding figures were even higher (36.3 contacts per year in the multimorbid and 15.9 in the non-multimorbid sample, respectively), whereby each additional chronic condition was associated with an increase of 2.3 contacts. In this same study, 5.7 different physicians were contacted in the multimorbid-sample compared to 3.5 in the non-multimorbid-sample, which is also slightly more compared to our analysis.

In accordance with our results, findings from a study by Starfield et al. [25] for a nonelderly population have shown that the number of visits to both, the primary care physicians and the specialists, was highly associated with the degree of comorbidity. Higher numbers of referrals to specialists were found in the Dutch survey as well [11]. Likewise, a higher burden of diseases was associated with higher referrals to specialists in U.S. Medicare beneficiaries aged 65 or older [12], even in patients with rather common chronic diseases that are generally in the purview of primary care physicians. Authors concluded that it might be more convenient and efficient for many conditions if specialists would serve as consultants to primary care physicians rather than undertake consultations directly with the patients.

The association between the number of diagnosis groups and hospital admissions among elderly has been reported previously for Sweden and Italy [8,9]. Increased hospitalisation rates in patients with multiple chronic conditions compared to none or one condition were further shown in a primary care-based Irish study [4], where each additional chronic condition was associated with a significant increase in the number of hospital admissions. Our findings on the higher length of stay were supported by the results of a Spanish longitudinal study [26], where the length of hospital stay has increased from 8.6 days to 12.1 days in patients with no versus patients with three or more comorbidities according to the Charlson Index Score.

### Health care costs

According to our analysis, mean total costs were 5.5 times higher in the multimorbid compared to the non-multimorbid sample. Each additional chronic condition was associated with increased total costs of CHF 2222 (US$ 2383) per year. We found an increase per each additional chronic condition on both, inpatient and outpatient costs, but the influence was greater on the latter.

An increase in costs with increasing number of chronic conditions was also reported by Glynn et al. [4], even though to a lesser extent. However, they did not include medication or paramedical costs, accounting for a great proportion of the difference, and costs were not calculated for each case individually. According to a systematic review by Lehnert et al. [10], total health care expenditures rose almost exponentially with the number of chronic conditions in several studies. For instance, in the U.S. study by Wolff et al. [2], mean expenditures in individuals without a chronic condition amounted to US$ 211 per capita compared to US$ 2394 for patients with two and to US$ 13'973 in patients with four or more chronic conditions. Studies included in the above review [10] that differentiated between inpatient and outpatient costs were all conducted in the U.S. and showed inconsistent results [27,28]. The high proportion of outpatient costs in our study equals the figure reported by the Swiss Federal Office of Public Health, where 47.6% of the total health care expenses in the statutory health insurance in 2011 accounted for outpatient services, 30.5% for inpatient services, and 21.9% accounted for medication costs [29]. The high standard deviations reported in our study are consistent with the standard deviations of the cited articles, suggesting great variance and heterogeneity in both samples.

### Influencing factors

The strong influence of nursing dependency on health care utilization reported by van den Bussche et al. [7] could also be found in the present study, and was found to be even stronger on the health care costs.

We discovered a significant age-gender interaction which influenced the results on health care utilization and costs. While consultations increased with increasing age in men, the increase in consultations declined with increasing age in women. Likewise, costs were constantly higher in men aged 70 or older compared to men aged 65 to 69 years, whereas costs in women decreased with increasing age. Several studies have found an impact of age and gender on the observed differences in health care utilization and costs between the multimorbid and non-multimorbid sample [7,11,17,24], whereas few reported no or small influences of age and gender [23]. In contrast to our findings, the effect of age on the increase in numbers of consultations was larger in females compared to males in the study by Glynn et al. [4]. However, their sample consisted of patients aged 50 years and older. In a comparative study by Fortin et al. [24], women were more likely to suffer from multiple chronic diseases than men in the general population, whereas more men than women were seen in the primary care setting. Differences in the severity of illness and in the health behaviour pattern might account for this discrepancy. This inverse association was also found by van den Bussche et al. [7]. The findings are supported by our results, where being female was associated with higher rates of multimorbidity and, at the same time, with lower costs.

As far as the insurance plan is concerned, accident coverage did not alter the results. This is probably due to the fact that the majority of the study population (98%) was insured and the difference between both samples may mainly be explained by age differences. The managed care model was associated with lower inpatient, but higher outpatient costs and a higher number of consultations, especially in primary care physicians. This is in line with a recent study by van der Heijden et al. [30], which showed lower number of hospitalisations for diabetic patients in a managed care model compared with usual care (0.7 versus 2.7), whereas the mean number of consultations was slightly higher (7.6 versus 6.1). One reason for this might be that the primary care physician is managing the patient more closely, thereby causing more consultations but lower hospitalisation rates. In contrast to our results, direct total costs were lower in their sample of managed care patients compared to usual care, whereas no influence on total costs resulted from our analysis. This difference might be related to the fact that our study sample was 10 years older.

The correlation between increased prevalence rates and increasing deprivation quintiles is supported by recent cross-sectional studies [3,17]. This finding is also consistent when other measures of socioeconomic status were used like education or free medical care eligibility [4,31]. One reason for the association between socioeconomic status and multimorbidity may be the fact, that the prevalence of risk factors for several chronic conditions is higher in populations with a lower socioeconomic status. Furthermore, we discovered that even though individuals living in communities with a lower purchasing power were more likely to suffer from multiple chronic conditions, lower purchasing power was associated with lower numbers of consultations and lower total costs. This is in contrast to findings from a German study [23], where sociodemographic variables did not seem to have an impact on the number of contacts with physicians. However, our results have to be interpreted with great caution as the analyses are not based on individual data regarding socioeconomic status. Furthermore, the effect sizes are very small. Therefore, further research is needed to deepen the knowledge in this field.

In summary, except for accident coverage all the covariates examined in the regression analyses have significantly influenced the relationship between multimorbidity, health care utilization and health care costs. However, as the differences in health care utilization and costs were rather small, their clinical significance is presumably quite small as well.

### Strengths and limitations

To the best of our knowledge, this is one of the few studies in Europe and the first in Switzerland investigating the association between multimorbidity and health care utilization and health care costs in the elderly. A large population with reliable information on health care utilization and health care costs was used to calculate the proportion of individuals with multimorbidity and its associated factors.

However, since clinical diagnosis were lacking, chronic conditions based on the PCG model were used to identify patients with multimorbidity. The prevalence estimates only rely on administrative data of treated conditions leading to potential misclassification. Although claim-based measures of multimorbidity are widely used [10,19], population-based estimates of the prevalence of multimorbidity were shown to be lower than estimates based on primary care samples [24]. Moreover, no standardized measure of multimorbidity exists for epidemiological surveys. As different countries and research groups are using different coding systems for chronic diseases, results cannot be compared directly with findings from other countries. However, the use of 22 prevalent chronic diseases in the present study fulfils the criteria set up by Fortin et al., who suggested using a list of at least 12 chronic diseases with a high burden in a given population [5].

Beyond that, our study findings are likely to be influenced by further factors, which are not included in the claims data, like the duration of consultations, the severity of illness or the social setting of the patients [9,13]. More particularly, living alone has been shown to be independently associated with hospital use [9].

In addition, the findings are subject to selection bias as data of privately insured patients could not be taken into account in these analyses. The exclusion of nursing home residents may have led to a further underestimation of the prevalence rate. Moreover, data concerning the health care costs are likely to be underestimated since a small proportion of claims invoices have not been sent to the health insurer or have not been gathered by them.

Finally, the cross-sectional design of the study does not allow drawing any causal conclusions.

### Implications

The high prevalence of multimorbidity challenges the present single-disease framework [3]. The fact that 76.6% of the elderly population suffer from multiple chronic diseases should urgently be addressed by health care professionals as well as policy makers, because the clinical needs differ from patients with only one chronic condition. Every additional consultation and/or contact may bring about new diagnosis, medication prescriptions and/or lifestyle advices which need to be understood and compiled with by the patients [32]. Likewise, the coordination of care (like the management of the patients’ medications) and the communication between physicians and allied health professionals poses major challenges. The risk of unnecessary replication of diagnostic tests, of adverse drug-drug or disease-drug interactions and of treatment errors rises in individuals with multiple chronic conditions [33]. This risk is particularly high in the elderly population because of the higher physical and mental demands which arise from treatment and drug regimes. Wolff et al. [2] have found a strong and positive relationship between the risk of avoidable hospital admissions or preventable complications and the number of chronic conditions in individuals aged 65 or older.

The current health care system is focussed on diseases rather than on patients. However, future costs cannot be calculated as the sum of the costs of single diseases [10]. Therefore, studies dealing with health care expenditures in patients with multiple chronic conditions are of great importance for each health care system. New evidence for safe and effective treatment plans and intervention programs are needed to manage the future societal and economic burden of multimorbidity. Case-management programs for patients with multiple diseases are currently being developed, although mostly in English speaking countries [34]. Our study makes a valuable contribution to the knowledge about the rise in health care utilization and costs with every additional chronic condition, indicating the urgent need for the development of such chronic care programs in Switzerland. In this population of elderly patients with multiple chronic diseases, management programs should consist of both, care by physicians as well as by nurses or allied health professionals, as the management of daily life, the transition between different health care settings, the adherence to physicians’ recommendations and the ongoing loss of self-government poses major challenges [33]. Future clinical trials should include older patients with multiple chronic conditions to set up guidelines for this growing population. Thereby, recommendations should incorporate the burden of treatment for patients and caregivers and allow for shared decision making aspects [33].

## Conclusions

In this sample of a Swiss community-dwelling population aged 65 or older, 76.6% had two or more chronic conditions. There is a considerable variation in health care utilization and total health care costs in individuals with and without multiple chronic conditions. Each additional chronic condition was associated with an increase of 3.2 consultations and increased costs of 33% per year. We found a significant interaction between age and gender. The huge burden of chronic diseases is crucial for health care policy debates about resource allocation. The expected increase in the prevalence of chronic conditions further intensifies the problem. Strategies for a better coordination of patients with multiple chronic conditions are urgently needed.

## Additional files

Additional file 1:
**Multiple linear regression model on the number of consultations by primary care physicians per year in an elderly population (≥ 65 years of age) (n=229493).**
Additional file 2:
**Multiple linear regression model on the number of consultations by specialists per year in an elderly population (≥ 65 years of age) (n=229493).**
Additional file 3:
**Multiple linear regression model on the inpatient health care costs per year in an elderly population (≥ 65 years of age) (n=229493).**
Additional file 4:
**Multiple linear regression model on the outpatient health care costs per year in an elderly population (≥ 65 years of age) (n=229493).**
