# Supplementary material for: Multimorbidity, health care utilization and costs in an elderly community-dwelling population: a claims data based observational study
Source: BMC Health Serv Res. 2015 Jan 22;15:23. doi: 10.1186/s12913-015-0698-2 (PMC4307623; doi:10.1186/s12913-015-0698-2)
Supplement: Additional file 1: — Multiple linear regression model on the number of consultations by primary care physicians per year in an elderly population (≥ 65 years of age) (n=229493). [file 12913_2015_698_MOESM1_ESM.doc]

Additional file 1: Multiple linear regression model on the number of consultations by primary care physicians per year in an elderly population (≥ 65 years of age) (n=229493).

| Number of consultations by primary care physicians | | |
| --- | --- | --- |
|  | B (95% CI) | *Sign.* |
| Age group by male gender |  |  |
| 65-69 (male) | 1.000 |  |
| 70-74 (male) | 1.048 (1.036 - 1.059) | *** |
| 75-79 (male) | 1.054 (1.041 - 1.067) | *** |
| 80-84 (male) | 1.084 (1.069 - 1.099) | *** |
| 85+ (male) | 1.109 (1.092 - 1.127) | *** |
| Age group by female gender |  |  |
| 65-69 (female) | 1.000 |  |
| 70-74 (female) | 1.033 (1.011 - 1.055) | ** |
| 75-79 (female) | 1.037 (1.014 - 1.060) | ** |
| 80-84 (female) | 1.042 (1.017 - 1.068) | *** |
| 85+ (female) | 1.052 (1.024 - 1.081) | *** |
| Number of chronic conditions | 1.128 (1.126 - 1.129) | *** |
| Linguistic region |  |  |
| German | 1.000 |  |
| French | 0.904 (0.897 - 0.912) | *** |
| Italian | 0.966 (0.957 - 0.976) | *** |
| Rhaeto-Romanic | 0.944 (0.887 - 1.005) |  |
| Purchasing power |  |  |
| 1 (high) | 1.000 |  |
| 2 | 0.994 (0.986 - 1.003) |  |
| 3 | 1.009 (1.000 - 1.018) | * |
| 4 | 1.031 (1.022 - 1.040) | *** |
| 5 (low) | 1.038 (1.029 - 1.047) | *** |
| Deductible class | 0.937 (0.929 - 0.945) | *** |
| Managed care | 1.061 (1.055 - 1.067) | *** |
| Accident coverage | 1.016 (0.996 - 1.036) |  |
| Nursing dependency | 1.123 (1.111 - 1.134) | *** |
| Corresponding consultations in 2012 | 1.736 (1.730 - 1.741) | *** |
| R^2^ | .541 |  |

*** p-value <0.001 ** p-value <0.01 * p-value <0.05
