# Supplementary material for: Multimorbidity, health care utilization and costs in an elderly community-dwelling population: a claims data based observational study
Source: BMC Health Serv Res. 2015 Jan 22;15:23. doi: 10.1186/s12913-015-0698-2 (PMC4307623; doi:10.1186/s12913-015-0698-2)
Supplement: Additional file 2: — Multiple linear regression model on the number of consultations by specialists per year in an elderly population (≥ 65 years of age) (n=229493). [file 12913_2015_698_MOESM2_ESM.doc]

Additional file 2: Multiple linear regression model on the number of consultations by specialists per year in an elderly population (≥ 65 years of age) (n=229493).

| Number of consultations by specialists | | |
| --- | --- | --- |
|  | B (95% CI) | *Sign.* |
| Age group by male gender |  |  |
| 65-69 (male) | 1.000 |  |
| 70-74 (male) | 1.046 (1.034 - 1.059) | *** |
| 75-79 (male) | 1.055 (1.041 - 1.069) | *** |
| 80-84 (male) | 1.049 (1.034 - 1.065) | *** |
| 85+ (male) | 1.002 (0.985 - 1.020) |  |
| Age group by female gender |  |  |
| 65-69 (female) | 1.000 |  |
| 70-74 (female) | 1.107 (1.081 - 1.133) | *** |
| 75-79 (female) | 1.069 (1.043 - 1.096) | *** |
| 80-84 (female) | 1.005 (0.979 - 1.033) |  |
| 85+ (female) | 0.927 (0.899 - 0.955) | *** |
| Number of chronic conditions | 1.103 (1.101 - 1.105) | *** |
| Linguistic region |  |  |
| German | 1.000 |  |
| French | 1.026 (1.018 - 1.035) | *** |
| Italian | 1.038 (1.027 - 1.049) | *** |
| Rhaeto-Romanic | 0.956 (0.892 - 1.025) |  |
| Purchasing power |  |  |
| 1 (high) | 1.000 |  |
| 2 | 0.970 (0.960 - 0.979) | *** |
| 3 | 0.921 (0.912 - 0.930) | *** |
| 4 | 0.889 (0.881 - 0.898) | *** |
| 5 (low) | 0.860 (0.851 - 0.868) | *** |
| Deductible class | 0.973 (0.963 - 0.982) | *** |
| Managed care | 1.004 (0.998 - 1.010) |  |
| Accident coverage | 0.982 (0.961 - 1.004) |  |
| Nursing dependency | 0.904 (0.894 - 0.915) | *** |
| Corresponding consultations in 2012 | 1.708 (1.702 - 1.713) | *** |
|  |  |  |
| R^2^ | .427 |  |

*** p-value <0.001 ** p-value <0.01 * p-value <0.05
