# Supplementary material for: Multimorbidity, health care utilization and costs in an elderly community-dwelling population: a claims data based observational study
Source: BMC Health Serv Res. 2015 Jan 22;15:23. doi: 10.1186/s12913-015-0698-2 (PMC4307623; doi:10.1186/s12913-015-0698-2)
Supplement: Additional file 3: — Multiple linear regression model on the inpatient health care costs per year in an elderly population (≥ 65 years of age) (n=229493). [file 12913_2015_698_MOESM3_ESM.doc]

Additional file 3: Multiple linear regression model on the inpatient health care costs per year in an elderly population (≥ 65 years of age) (n=229493).

| Inpatient health care costs | | |
| --- | --- | --- |
|  | B (95% CI) | *Sign.* |
| Age group by male gender |  |  |
| 65-69 (male) | 1.000 |  |
| 70-74 (male) | 1.026 (1.001 - 1.052) | * |
| 75-79 (male) | 1.061 (1.033 - 1.090) | *** |
| 80-84 (male) | 1.079 (1.047 - 1.112) | *** |
| 85+ (male) | 0.971 (0.938 - 1.006) |  |
| Age group by female gender |  |  |
| 65-69 (female) | 1.000 |  |
| 70-74 (female) | 0.885 (0.843 - 0.928) | *** |
| 75-79 (female) | 0.856 (0.814 - 0.900) | *** |
| 80-84 (female) | 0.800 (0.757 - 0.844) | *** |
| 85+ (female) | 0.676 (0.636 - 0.718) | *** |
| Number of chronic conditions | 1.219 (1.215 - 1.222) | *** |
| Linguistic region |  |  |
| German | 1.000 |  |
| French | 0.828 (0.814 - 0.843) | *** |
| Italian | 0.932 (0.911 - 0.953) | *** |
| Rhaeto-Romanic | 0.980 (0.852 - 1.127) |  |
| Purchasing power |  |  |
| 1 (high) | 1.000 |  |
| 2 | 0.993 (0.974 - 1.013) |  |
| 3 | 0.980 (0.961 - 0.999) | * |
| 4 | 0.976 (0.957 - 0.995) | * |
| 5 (low) | 0.960 (0.941 - 0.979) | *** |
| Deductible class | 1.140 (1.119 - 1.162) | *** |
| Managed care | 0.981 (0.969 - 0.994) | ** |
| Accident coverage | 0.984 (0.942 - 1.028) |  |
| Nursing dependency | 3.314 (3.237 - 3.392) | *** |
| Inpatient health care costs in 2012 | 1.098 (1.094 - 1.103) | *** |
|  |  |  |
| R^2^ | .156 |  |

*** p-value <0.001 ** p-value <0.01 * p-value <0.05
