# Supplementary material for: Multimorbidity, health care utilization and costs in an elderly community-dwelling population: a claims data based observational study
Source: BMC Health Serv Res. 2015 Jan 22;15:23. doi: 10.1186/s12913-015-0698-2 (PMC4307623; doi:10.1186/s12913-015-0698-2)
Supplement: Additional file 4: — Multiple linear regression model on the outpatient health care costs per year in an elderly population (≥ 65 years of age) (n=229493). [file 12913_2015_698_MOESM4_ESM.doc]

Additional file 4: Multiple linear regression model on the outpatient health care costs per year in an elderly population (≥ 65 years of age) (n=229493).

| Outpatient health care costs | | |
| --- | --- | --- |
|  | B (95% CI) | *Sign.* |
| Age group by male gender |  |  |
| 65-69 (male) | 1.000 |  |
| 70-74 (male) | 1.076 (1.060 - 1.092) | *** |
| 75-79 (male) | 1.084 (1.066 - 1.102) | *** |
| 80-84 (male) | 1.053 (1.034 - 1.072) | *** |
| 85+ (male) | 1.032 (1.010 - 1.054) | ** |
| Age group by female gender |  |  |
| 65-69 (female) | 1.000 |  |
| 70-74 (female) | 1.048 (1.018 - 1.079) | ** |
| 75-79 (female) | 0.994 (0.964 - 1.025) |  |
| 80-84 (female) | 0.936 (0.906 - 0.968) | *** |
| 85+ (female) | 0.893 (0.861 - 0.926) | *** |
| Number of chronic conditions | 1.256 (1.253 - 1.259) | *** |
| Linguistic region |  |  |
| German | 1.000 |  |
| French | 1.006 (0.995 - 1.017) |  |
| Italian | 0.993 (0.980 - 1.007) |  |
| Rhaeto-Romanic | 1.007 (0.925 - 1.095) |  |
| Purchasing power |  |  |
| 1 (high) | 1.000 |  |
| 2 | 0.956 (0.945 - 0.967) | *** |
| 3 | 0.914 (0.903 - 0.925) | *** |
| 4 | 0.880 (0.869 - 0.890) | *** |
| 5 (low) | 0.844 (0.834 - 0.854) | *** |
| Deductible class | 0.865 (0.855 - 0.875) | *** |
| Managed care | 1.017 (1.010 - 1.025) | *** |
| Accident coverage | 0.992 (0.966 - 1.019) |  |
| Nursing dependency | 2.046 (2.017 - 2.076) | *** |
| Outpatient health care costs in 2012 | 1.508 (1.503 - 1.513) | *** |
|  |  |  |
| R^2^ | .551 |  |

*** p-value <0.001 ** p-value <0.01 * p-value <0.05
